# Supplementary material for: Endothelial pannexin-1 channels modulate macrophage and smooth muscle cell activation in abdominal aortic aneurysm formation
Source: Nat Commun. 2022 Mar 21;13:1521. doi: 10.1038/s41467-022-29233-4 (PMC8938517; doi:10.1038/s41467-022-29233-4)
Supplement: Supplementary file 1 — Supplementary Information [file 41467_2022_29233_MOESM1_ESM.pdf]

Supplementary Information Inventory

Endothelial pannexin-1 channels modulate macrophage and smooth muscle cell activation in abdominal aortic aneurysm formation

Amanda C. Filiberto[1†], Michael D. Spinosa[2†], Craig T. Elder[1], Gang Su[1], Victoria Leroy[1], Zachary Ladd[1], Guanyi Lu[1], J. Hunter Mehaffey[2], Morgan D. Salmon[2], Robert B. Hawkins[2], Kodi S. Ravichandran[3], Brant E. Isakson[4], Gilbert R. Upchurch, Jr.[1], & Ashish K. Sharma[1*]

[1]Department of Surgery, University of Florida, Gainesville, FL

[2]Department of Surgery, University of Virginia, Charlottesville, VA

[3]Department of Microbiology, Immunology and Cancer Biology, University of Virginia, Charlottesville, VA

[4]Department of Molecular Physiology and Biological Physics, University of Virginia, Charlottesville, VA

† Equal contribution

Correspondence to: Email: ashish.sharma@surgery.ufl.edu (A.K.S.)

Contents:

1. Supplementary Figures
    a. Supplementary Figure 1
    b. Supplementary Figure 2
    c. Supplementary Figure 3
    d. Supplementary Figure 4
    e. Supplementary Figure 5
    f. Supplementary Figure 6
    g. Supplementary Figure 7
    h. Supplementary Figure 8

2. Source Data File in .xlsx format (separate)

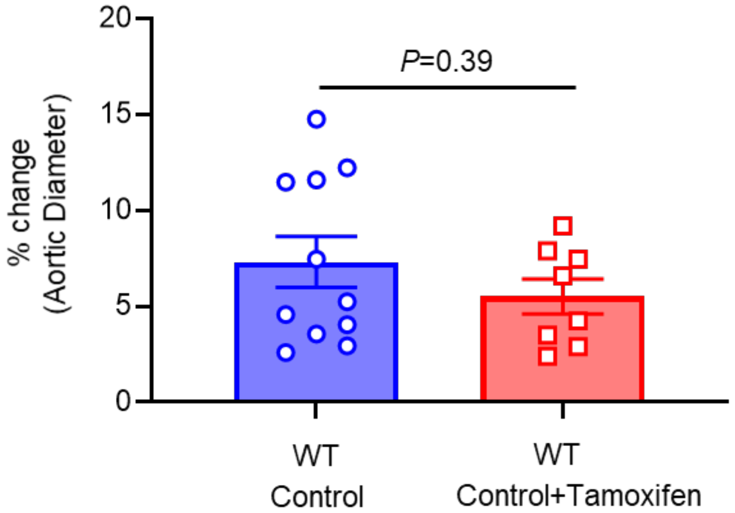

Supplementary Fig. 1 Tamoxifen treatment does not alter the aortic diameter compared to deactivated elastase-treated (control) mice on day 14. n=8-11/group. Data is represented as mean values ± SEM and comparative statistical analyses was done by two-tailed t-test. Source data are provided as a Source Data file.

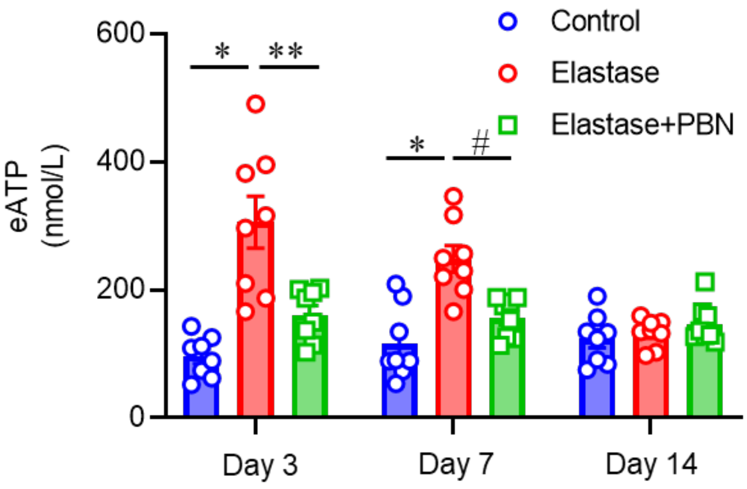

Supplementary Fig. 2 A significant increase in extracellular (e)-ATP content was observed in plasma of elastase-treated mice compared to controls. PBN-treated mice displayed a significant attenuation of eATP compared to elastase-treated mice.**P*<0.0001; ***P*=0.001; $^{\#}P$=0.004; n=8 mice/group. Data is represented as mean values ± SEM and comparative statistical analyses was done by one-way ANOVA followed by multiple comparisons. Source data are provided as a Source Data file.

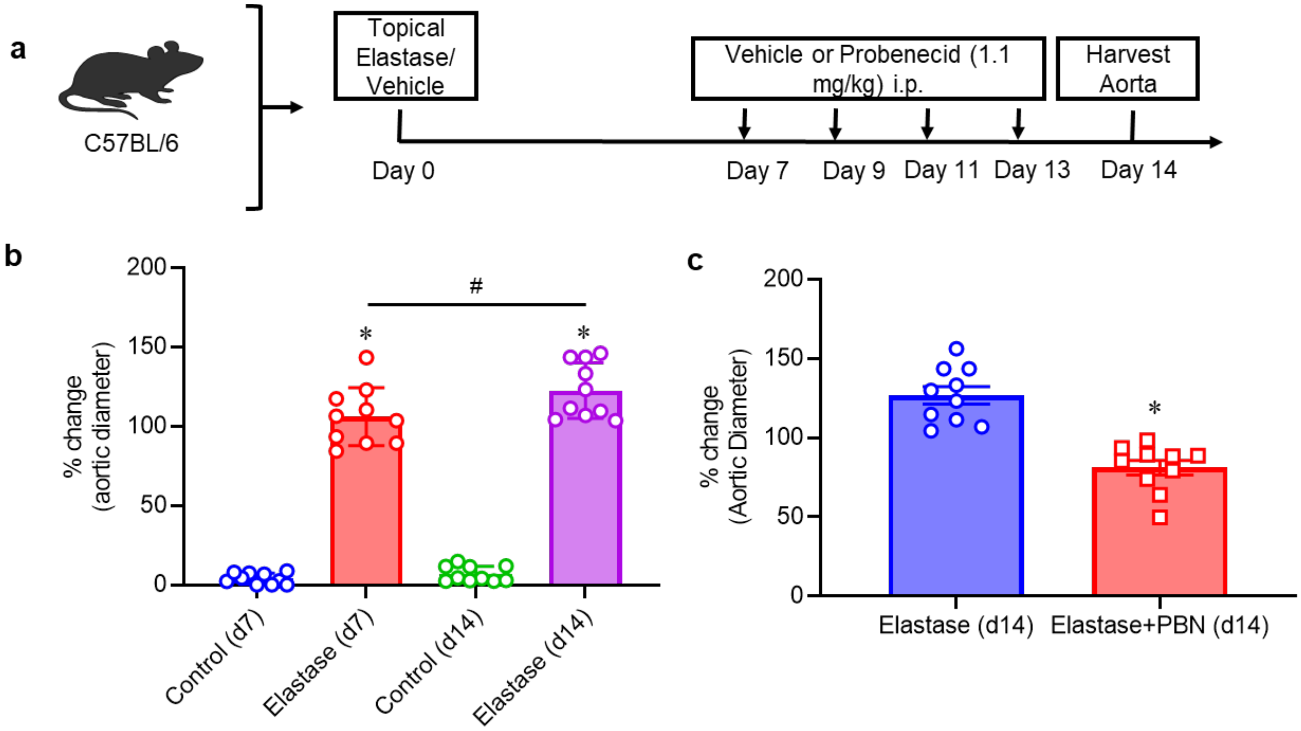

Supplementary Fig. 3 a Using the elastase AAA murine model, the protective role of Probenecid (PBN) administered **after** the formation of AAAs on day 7 was investigated. **b** AAA formation in elastase-treated WT mice was significantly increased compared to respective controls on days 7 and 14, respectively. *$P<0.0001$ vs. respective controls; #$P=0.03$; n=8-10/group. Data is represented as mean values ± SEM and comparative statistical analyses was done by one-way ANOVA. **c** Treatment with PBN (after day 7) significantly attenuated aortic diameter in elastase-treated mice on day 14. *$P=0.0001$; n=10/group. Data is represented as mean values ± SEM and comparative statistical analyses was done by two-tailed t-test. Source data are provided as a Source Data file.

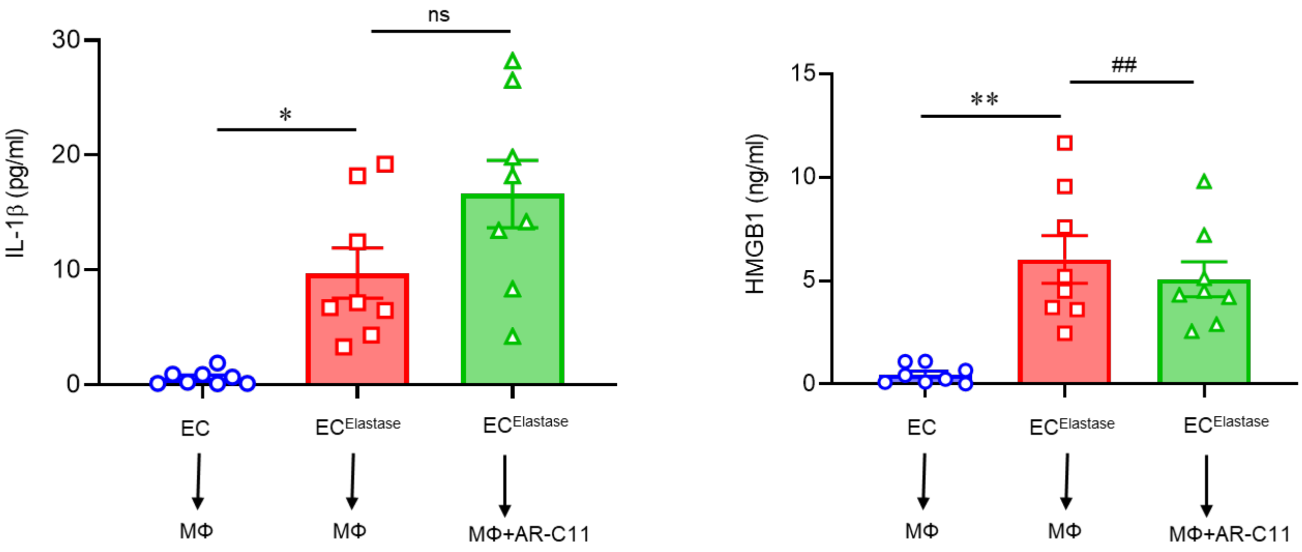

Supplementary Fig. 4 P2Y2 inhibition of macrophages fails to mitigate cytokine secretion induced by EC-dependent Panx1/ATP signaling. CMT from elastase-treated ECs to macrophages induces a significant upregulation of IL-1β and HMGB1 secretion, which was not blocked by pretreatment of macrophages with P2Y2 inhibitor (AR-C11; 10μM). **P*=0.01; ***P*=0.0003; ns, not significant, *P*=0.07; $^{\#\#}$*P*=0.69; n=8/group. Data is represented as mean values ± SEM and comparative statistical analyses was done by one-way ANOVA. Source data are provided as a Source Data file.

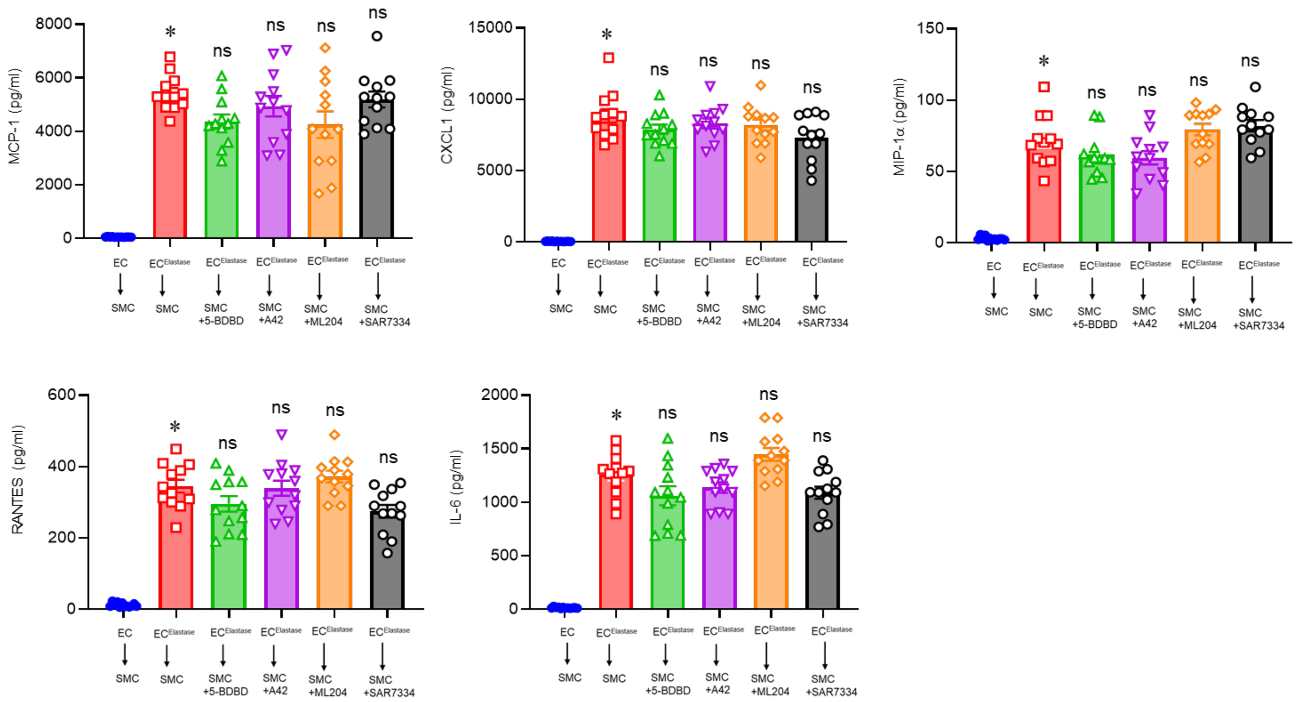

Supplementary Fig. 5 P2X4, TRPV1, TRPC4 or TRPC6 inhibition of SMCs does not attenuate cytokine secretion. Activation of SMCs by CMT from elastase-treated ECs results in a multifold increase in proinflammatory cytokine production which was not inhibited by pretreatment with either P2X4 (5-BDBD; 50μM), TRPV1 (A42), TRPC4 (ML204) or TRPC6 (SAR73) inhibitors (1μM each). *P<0.001 vs. control (EC→SMC); ns, not significant vs. EC(elastase)→SMC; n=12/group. Data is represented as mean values ± SEM and comparative statistical analyses was done by one-way ANOVA. Source data are provided as a Source Data file.

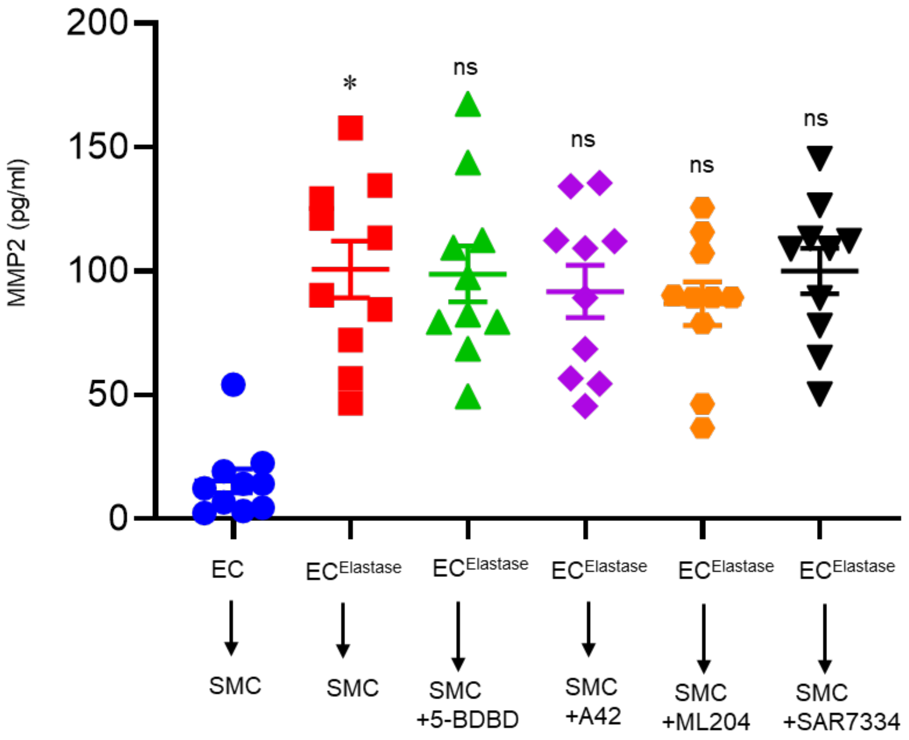

Supplementary Fig. 6 P2X4, TRPV1, TRPC4 or TRPC6 inhibition of SMCs does not attenuate MMP2 activity. Activation of SMCs by CMT from elastase-treated ECs results in a multifold increase in MMP2 activity which was not inhibited by pretreatment with either P2X4 (5-BDBD; 50μM), TRPV1 (A42), TRPC4 (ML204) or TRPC6 (SAR73) inhibitors (1μM each). **P*=0.0001; ns, not significant, *P*=0.9 vs. EC(elastase)→SMC; n=8/group. Data is represented as mean values ± SEM and comparative statistical analyses was done by one-way ANOVA. Source data are provided as a Source Data file.

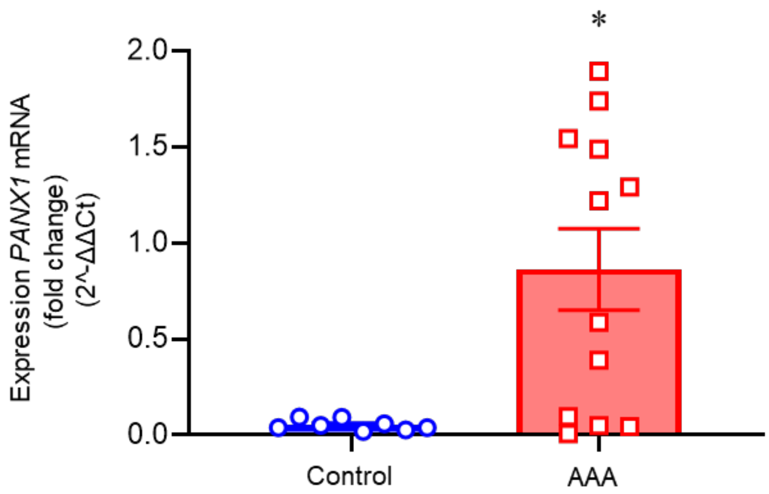

Supplementary Fig. 7 *PANX1* mRNA expression is increased in AAA patients. A multi-fold increase in *PANX1* mRNA expression was observed in aortic tissue from AAA patients compared to controls. **P*=0.01; n=8-12/group. Data is represented as mean values ± SEM and comparative statistical analyses was done by two-tailed t-test. Source data are provided as a Source Data file.

Kaplan-Meier Survival Curve

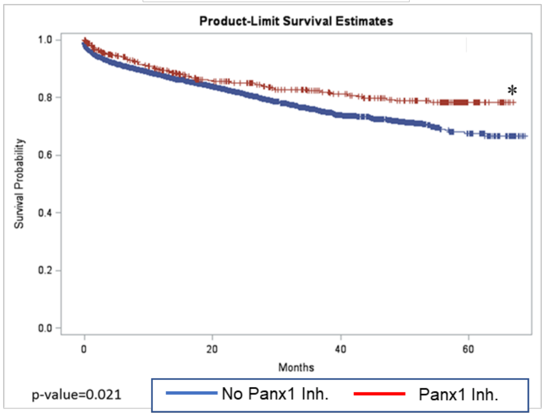

Cox Proportional Hazard Model

Parameter	Hazards Ratio	p-value
**Age (years)**	1.05	<0.0001
**Sex (Female)**	0.93	0.357
**Race (White)**	0.94	0.605
**Tobacco Use**	1.57	0.014
**Diabetes Mellitus**	1.05	0.579
**Hypertension**	1.23	0.456
**Chronic Obstructive Pulmonary Disease**	1.76	<0.0001
**Peripheral Vascular Disease**	1.15	0.133
**Congestive Heart Failure**	1.78	<0.0001
**Cancer**	1.48	<0.0001
**Stroke**	2.89	<0.0001
**Operative Repair**	0.68	<0.0001
**Panx1 Inhibitors**	0.65	0.001

Univariate differences between groups

Variable	Panx1 Inhibitors (n=440)	No Panx1 Inhibitors (n=4109)	p value
**Age (years)**	66 ± 16	67 ± 16	0.198
**Sex (Male)**	299 (68.0%)	2773 (67.5%)	0.842
**Race (White)**	377 (85.7%)	3575 (87.0%)	0.435
**Tobacco Use**	74 (16.8%)	620 (15.1%)	0.176
**Diabetes Mellitus**	116 (26.4%)	868 (21.1%)	0.011
**Hypertension**	378 (85.9%)	3558 (86.6%)	0.737
**Chronic Obstructive Pulmonary Disease**	103 (23.4%)	892 (21.7%)	0.412
**Peripheral Vascular Disease**	82 (18.6%)	720 (17.5%)	0.560
**Congestive Heart Failure**	134 (30.5%)	605 (14.7%)	<0.001
**Cancer**	85 (19.3%)	771 (18.8%)	0.777
**Stroke**	27 (6.1%)	266 (6.5%)	0.784
**Operative Repair**	19 (4.3%)	227 (5.5%)	0.288
**Probenecid**	141 (3.1%)	-	
**Spironolactone**	313 (6.9%)	-	

Supplementary Fig. 8 Panx1 inhibitors are associated with decreased mortality in aortic aneurysm patients. Retrospective analysis of human aortic aneurysm patient data demonstrates a significantly lower risk of mortality after 5-years in patients who were receiving Probenecid or Spironolactone (Panx1 inhibitors) compared to aortic aneurysm patients without these medications. Kaplan-Meier survival analysis and Cox Proportional Hazards model was utilized to compare unadjusted and risk-adjusted long-term survival respectively. **P*=0.02.
