# Supplementary material for: Endothelial pannexin-1 channels modulate macrophage and smooth muscle cell activation in abdominal aortic aneurysm formation
Source: Nat Commun. 2022 Mar 21;13:1521. doi: 10.1038/s41467-022-29233-4 (PMC8938517; doi:10.1038/s41467-022-29233-4)
Supplement: Supplementary file 3 — Reporting Summary [file 41467_2022_29233_MOESM3_ESM.pdf]

nature portfolio

Corresponding author(s): NCOMMS-21-26510A-Z
Ashish K. Sharma

Last updated by author(s): Feb 13, 2022

Reporting Summary

Nature Portfolio wishes to improve the reproducibility of the work that we publish. This form provides structure for consistency and transparency in reporting. For further information on Nature Portfolio policies, see our Editorial Policies and the Editorial Policy Checklist.

Statistics

For all statistical analyses, confirm that the following items are present in the figure legend, table legend, main text, or Methods section.

n/a	Confirmed	
☐	☒	The exact sample size (*n*) for each experimental group/condition, given as a discrete number and unit of measurement
☐	☒	A statement on whether measurements were taken from distinct samples or whether the same sample was measured repeatedly
☐	☒	The statistical test(s) used AND whether they are one- or two-sided<br>*Only common tests should be described solely by name; describe more complex techniques in the Methods section.*
☐	☒	A description of all covariates tested
☐	☒	A description of any assumptions or corrections, such as tests of normality and adjustment for multiple comparisons
☐	☒	A full description of the statistical parameters including central tendency (e.g. means) or other basic estimates (e.g. regression coefficient) AND variation (e.g. standard deviation) or associated estimates of uncertainty (e.g. confidence intervals)
☐	☒	For null hypothesis testing, the test statistic (e.g. *F*, *t*, *r*) with confidence intervals, effect sizes, degrees of freedom and *P* value noted<br>*Give P values as exact values whenever suitable.*
☒	☐	For Bayesian analysis, information on the choice of priors and Markov chain Monte Carlo settings
☒	☐	For hierarchical and complex designs, identification of the appropriate level for tests and full reporting of outcomes
☒	☐	Estimates of effect sizes (e.g. Cohen's *d*, Pearson's *r*), indicating how they were calculated

Our web collection on statistics for biologists contains articles on many of the points above.

Software and code

Policy information about availability of computer code

Data collection	Video micrometry (Nikon SMZ-25)<br>Immunohistochemistry (AxioCam MrC camera, Carl Zeiss)<br>BioRad C1000 Thermal Cycler<br>BioRad CFX96 Real-Time System<br>BioRad iMark microplate reader<br>Luminex 200<br>Olympus FluoView 1000
Data analysis	NIS Elements D5.10.01<br>Bio-Rad CFX Manager 3.1<br>AxioCam software version 4.6<br>Image-Pro Plus version 7.0<br>GraphPad Prism 6 software<br>SAS version 9.4 software<br>Microplate manager version 6.3<br>Bioplex manager 6.2

For manuscripts utilizing custom algorithms or software that are central to the research but not yet described in published literature, software must be made available to editors and reviewers. We strongly encourage code deposition in a community repository (e.g. GitHub). See the Nature Portfolio guidelines for submitting code & software for further information.

Data

Policy information about availability of data

All manuscripts must include a data availability statement. This statement should provide the following information, where applicable:

- Accession codes, unique identifiers, or web links for publicly available datasets
- A description of any restrictions on data availability
- For clinical datasets or third party data, please ensure that the statement adheres to our policy

All data supporting the findings of this study are available within the Article, Supplementary Information or Source Data file. Source data are provided with this paper.

Field-specific reporting

Please select the one below that is the best fit for your research. If you are not sure, read the appropriate sections before making your selection.

☒ Life sciences ☐ Behavioural & social sciences ☐ Ecological, evolutionary & environmental sciences

For a reference copy of the document with all sections, see nature.com/documents/nr-reporting-summary-flat.pdf

Life sciences study design

All studies must disclose on these points even when the disclosure is negative.

Sample size	Sample sizes were determined based on our studies and previous experimental experience with similar assays (Circulation; 2012; 126:S38-45 and FASEB; 2020:34(7):9787-9801)
Data exclusions	In case of mice, when the mice were dead unexpectedly due to the technical issues during the aneurysm formation, the data of the individual mice were excluded. No data were excluded from in vitro studies or human studies.
Replication	Each experiment was run in technical duplicates or triplicates, and the biological experiments were performed in independent replicates. The experiments were usually conducted at least three times independently to ensure reproducibility of the data and for statistical analysis. Individual values are shown in each figure.
Randomization	Mice were matched into groups according to the genotype, age and body weight, and then the mice were allocated and selected randomly. The investigators were blinded to group allocation during data collection using the mouse identification number, which did not specify the allocated group. In vitro studies, samples were randomly allocated into the different treatment group.
Blinding	During data collection and sample analysis using analysis equipment, the investigators were blinded to the mouse genotype, the group allocation, clinical backgrounds using the numerical code, which did not specify the allocated group. When the mice were treated with the specific chemicals, the investigators were not blinded because the drugs had to be administrated to the same mouse. The investigators were eventually blinded to all group allocations using the numerical code during data collection and/or analyses.

Reporting for specific materials, systems and methods

We require information from authors about some types of materials, experimental systems and methods used in many studies. Here, indicate whether each material, system or method listed is relevant to your study. If you are not sure if a list item applies to your research, read the appropriate section before selecting a response.

Materials & experimental systems

n/a	Involved in the study	
☐	☒	Antibodies
☐	☒	Eukaryotic cell lines
☒	☐	Palaeontology and archaeology
☐	☒	Animals and other organisms
☐	☒	Human research participants
☒	☐	Clinical data
☒	☐	Dual use research of concern

Methods

n/a	Involved in the study	
☒	☐	ChIP-seq
☒	☐	Flow cytometry
☒	☐	MRI-based neuroimaging

Antibodies

Antibodies used	Antibodies for immunohistochemical staining:<br>Anti-mouse Mac2 for macrophages (1:10,000; catalog no. CL8942AP, Cedarlane Laboratories, Burlington, ON, Canada)<br>Anti-mouse neutrophils for polymorphonuclear neutrophils (PMNs) (1:10,000; catalog no. MCA771GA, AbD Serotec, Oxford, UK)

	Anti-mouse a-smooth muscle actin (a-SMA; 1:1000; A5691-2ml; 1A-4 monoclonal, Sigma Aldrich, St. Louis, MO)<br>Anti-mouse Panx1 antibody (1:50; Cat No. HPA016930, Sigma Aldrich, St. Louis, MO)<br>Anti-rabbit secondary F(ab)'2 antibody coupled to Alexa Fluor 568 (A-11011, 1:400; Life Technologies, Carlsbad, CA)<br>Elastin staining (Verhoeff Van Gieson elastin stain, catalog no. 25089-1; Poly Sciences, Warrington, PA)<br>Anti-mouse CD3 for T cells (1:500; catalog no. SC-20047; Santa Cruz Biotech, Dallas, TX)
Validation	As per the manufacturer's product information, mouse monoclonal anti-a-smooth muscle actin antibody (A5691, Sigma Aldrich) detects endogenous a-smooth muscle actin by immunostaining of frozen and paraffin tissue sections. Anti-actin, a-smooth muscle cell antibody is validated as it specifically recognizes the a-smooth muscle actin isoform (42 kDa) in ELISA and immunoblotting.<br>As per the manufacturer's product information, rat anti-mouse Ly6G antibody (AbD Serotec, Oxford) detects endogenous mouse Ly6B.2 by immunostaining of frozen and paraffin tissue sections. This antibody has been validated by successfully using for the depletion of mature neutrophils in vivo.<br>As per the manufacturer's product information, rabbit anti-Mac-2 antibody (CL8942AP; Cedarlane laboratories) detects endogenous mouse LGALS3 by immunostaining of frozen and paraffin tissue sections. This antibody specifically binds the mouse Mac-2 antigen (Galectin-3) as it recognizes a 32,000 dalton surface antigen found on mouse macrophages.<br>As per the manufacturer's product information, anti-CD3 antibody (PC3/188A; Santa Cruz Biotech) is recommended for detection of CD3 of mouse origin by immunohistochemistry. This antibody has been validated by western blot analysis of CD3 expression in whole cell lysates.<br>As per the manufacturer's product information, anti-Panx1 antibody (Sigma Aldrich) produced in rabbit detects pannexin-1 in immunohistochemistry analyses in tissue and cell lines. This antibody has been validated by IHC tissue array, protein array and western blot analyses.<br>As per the manufacturer's product information, anti-rabbit secondary antibody coupled to Alexa Fluor 568 (Life Technologies) has been validated for use in immunostaining as a secondary antibody. This antibody has been validated by IHC and flow cytometry analyses.<br>As per the manufacturer's product information, Van Gieson's solution (PolySciences Inc.) has been validated for use in immunostaining of elastic tissue by Verhoeff's method. Formalin-fixed, paraffin-embedded sections are stained blue-black for elastic fibers.

Eukaryotic cell lines

Policy information about cell lines

Cell line source(s)	C57BL/6 murine primary aortic endothelial cells (C57-6052; Cell Biologics, Chicago, IL)
Authentication	As per the manufacturer, C57BL/6 Mouse Primary Aortic Endothelial Cells are tested for expression of markers using antibody, VE-cadherin (CD144, VE-cadherin Antibody, C-19, sc6458, Santa Cruz); AF1002 (R&D System) or CD31/PECAM-1 (Purified Rat Anti-Mouse CD31, Catalog No. 553370, BD) by immunofluorescence staining or FACS.
Mycoplasma contamination	As per the manufacturer, C57BL/6 Mouse Primary Aortic Endothelial Cells are negative for mycoplasma.
Commonly misidentified lines<br>(See ICLAC register)	No commonly misidentified cell lines were used.

Animals and other organisms

Policy information about studies involving animals; ARRIVE guidelines recommended for reporting animal research

Laboratory animals	This study used 8–12-wk-old male C57BL/6 WT mice (Jackson Laboratory, Bar Harbor, ME). The inducible, endothelial-specific Panx1 knockout mice, VE-Cad-CreERT2+Panx1fl/fl mice (EC-Panx1-/-), were generated by crossing vascular endothelial (VE)-Cad-CreERT2+ mice with Panx1fl/fl mice, as previously described and characterized (Nature Communications; 2015: 6, 7965). The inducible smooth muscle cell-specific Panx1 knockout, SMMHC-CreERT2+/Panx1fl/fl (SMC-Panx1-/-) mice were generated using SM-MHC-CreERT2+ mice with Panx1fl/fl mice, as previously described (Sci Signal; 2015; 8,ra17). C57BL/6 (Stock No. 000664) and ApoE-/- mice (Stock No. 002052) were obtained from Jackson laboratories (Bar Harbor, Maine). Mice were housed in a pathogen-free animal facility under a 12 h light/dark cycle at constant temperature and humidity, and fed standard rodent chow and water ad libitum.
Wild animals	The study did not involve wild animals.
Field-collected samples	The study did not involve samples collected from the field.
Ethics oversight	All animal studies were reviewed and approved by the University of Florida's Institutional Animal Care and Use Committee (protocol #202110051).

Note that full information on the approval of the study protocol must also be provided in the manuscript.

Human research participants

Policy information about studies involving human research participants

Population characteristics	Collection of human aortic tissue was approved by the Institutional Review Board, University of Florida (protocol # 13178). Preoperative consent was obtained from all patients and no participant compensation was provided. AAA tissue from male patients was resected during open surgical AAA repair, and abdominal aortic tissue was obtained from transplant donor patients as controls (total n=20; average age: 63 years). For the clinical data analyses, the University of Virginia

	institutional review board approved this study (protocol #17900). All adults (male and female; average age 67 years) with the diagnosis of aortic aneurysm using International Diagnosis Codes (ICD 9 or 10) between 1995 and 2015 were reviewed. Patients were not discriminated according to age, genotype information, or past/current diagnosis.
Recruitment	The patients were recruited consecutively at hospitalization and outpatient-clinic and consent was obtained from each patient. Aortic samples were collected during perioperative surgery and control aortic tissue was obtained from separate individuals undergoing transplant surgeries. All adult AAA patients and organ donors were recruited after informed consent authorization to participate in the research study. No bias or coercing occurred during or after recruitment and consent to avoid any impact on results, and participation in the study was completely voluntary.
Ethics oversight	The protocol of the human aortic tissue was approved by the Institutional Review Board, University of Florida. The clinical data retrospective analyses protocol was approved by Institutional Review Board, University of Virginia.

Note that full information on the approval of the study protocol must also be provided in the manuscript.
